# Analysis of sleep disorders under pain using an optogenetic tool: possible involvement of the activation of dorsal raphe nucleus-serotonergic neurons

**DOI:** 10.1186/1756-6606-6-59

**Published:** 2013-12-26

**Authors:** Hisakatsu Ito, Makoto Yanase, Akira Yamashita, Chigusa Kitabatake, Asami Hamada, Yuki Suhara, Michiko Narita, Daigo Ikegami, Hiroyasu Sakai, Mitsuaki Yamazaki, Minoru Narita

**Affiliations:** 1 Department of Pharmacology, Hoshi University School of Pharmacy and Pharmaceutical Sciences, 2-4-41 Ebara, Shinagawa-ku, Tokyo 142-8501, Japan; 2 Department of Anesthesiology, Graduate School of Medical and Pharmaceutical Sciences for Education, Toyama University, 2630 Sugitani, Toyama 930-0194, Japan

**Keywords:** Optogenetics, Electroencephalogram, Dorsal raphe nucleus, Neuropathic pain, Sleep

## Abstract

**Background:**

Several etiological reports have shown that chronic pain significantly interferes with sleep. Inadequate sleep due to chronic pain may contribute to the stressful negative consequences of living with pain. However, the neurophysiological mechanism by which chronic pain affects sleep-arousal patterns is as yet unknown. Although serotonin (5-HT) was proposed to be responsible for sleep regulation, whether the activity of 5-HTergic neurons in the dorsal raphe nucleus (DRN) is affected by chronic pain has been studied only infrequently. On the other hand, the recent development of optogenetic tools has provided a valuable opportunity to regulate the activity in genetically targeted neural populations with high spatial and temporal precision. In the present study, we investigated whether chronic pain could induce sleep dysregulation while changing the activity of DRN-5-HTergic neurons. Furthermore, we sought to physiologically activate the DRN with channelrhodopsin-2 (ChR2) to identify a causal role for the DRN-5-HT system in promoting and maintaining wakefulness using optogenetics.

**Results:**

We produced a sciatic nerve ligation model by tying a tight ligature around approximately one-third to one-half the diameter of the sciatic nerve. In mice with nerve ligation, we confirmed an increase in wakefulness and a decrease in non-rapid eye movement (NREM) sleep as monitored by electroencephalogram (EEG). Microinjection of the retrograde tracer fluoro-gold (FG) into the prefrontal cortex (PFC) revealed several retrogradely labeled-cells in the DRN. The key finding of the present study was that the levels of 5-HT released in the PFC by the electrical stimulation of DRN neurons were significantly increased in mice with sciatic nerve ligation. Using optogenetic tools in mice, we found a causal relationship among DRN neuron firing, cortical activity and sleep-to-wake transitions. In particular, the activation of DRN-5-HTergic neurons produced a significant increase in wakefulness and a significant decrease in NREM sleep. The duration of NREM sleep episodes was significantly decreased during photostimulation in these mice.

**Conclusions:**

These results suggest that neuropathic pain accelerates the activity of DRN-5-HTergic neurons. Although further loss-of-function experiments are required, we hypothesize that this activation in DRN neurons may, at least in part, correlate with sleep dysregulation under a neuropathic pain-like state.

## Background

It is generally acknowledged that neuropathic pain is extremely difficult to treat, and a major factor that affects outcomes is the presence of comorbidities, such as poor sleep and mood disorders. In clinical studies, most patients began to have difficulties with sleep after they began experiencing chronic pain [1,2]. However, the neurophysiological mechanism by which chronic pain affects sleep/arousal patterns is as yet unknown.

Serotonin (5-HT) was initially proposed to be responsible for the initiation and maintenance of sleep [3]. The firing rate of 5-HT-containing dorsal raphe nucleus (DRN) neurons decreases during slow wave sleep relative to that in wakefulness [4]. 5-HTergic neurons in the DRN fire tonically and regularly at 3–5 Hz in wakefulness, fire less during non–rapid eye movement (NREM) sleep, and are silent during rapid eye movement (REM) sleep [5-9]. Therefore, the DRN is a 5-HTergic brainstem structure that is thought to be important for promoting arousal.

Some neurons in the DRN respond to nociceptive stimuli. Noxious stimulation caused changes in the electrical activity of DRN neurons [10]. This concept was confirmed by the 2-deoxy-D-glucose [11] and c-fos [12] methods. However, whether the activity of 5-HTergic neurons in the DRN is affected by chronic pain has been studied only infrequently. In a previous study, 5-HT transporter expression was shown to be significantly increased in the DRN of sciatic nerve-ligated rats [13]. To investigate the influence of neuropathic pain on DRN 5-HTergic neurons, we performed an in vivo dialysis study. We found that the 5-HT levels in the prefrontal cortex (PFC) were significantly increased in nerve-ligated mice compared to those in sham-operated mice after electrical stimulation of the DRN.

New tools are needed to selectively manipulate the discharge activities of the DRN in freely moving mice at time-scales that are relevant to natural sleep-wake events. The recent development of an optogenetic tool [14,15] has provided a valuable opportunity to regulate the activity in genetically targeted neural populations with high spatial and temporal precision [16-19]. Optogenetics describes the now widespread use of microbial opsins [20] or related tools, that can be activated by illumination to manipulate cells even within intact tissue or behaving animals [21-23]. We stimulated DRN neurons with channelrhodopsin-2 (ChR2; a cation channel that is sensitive to 473 nm blue light) [17]. We found that mice showed an increase in the mean time of wakefulness, and decreases in the mean time and duration of NREM sleep, during optical stimulation of the DRN. To our knowledge, no previous reports have described the results obtained by stimulation of the DRN, which suggest a potential mechanism for the sleep disturbance induced by neuropathic pain.

## Results

### Thermal hyperalgesia induced by sciatic nerve ligation

**Figure 1:**
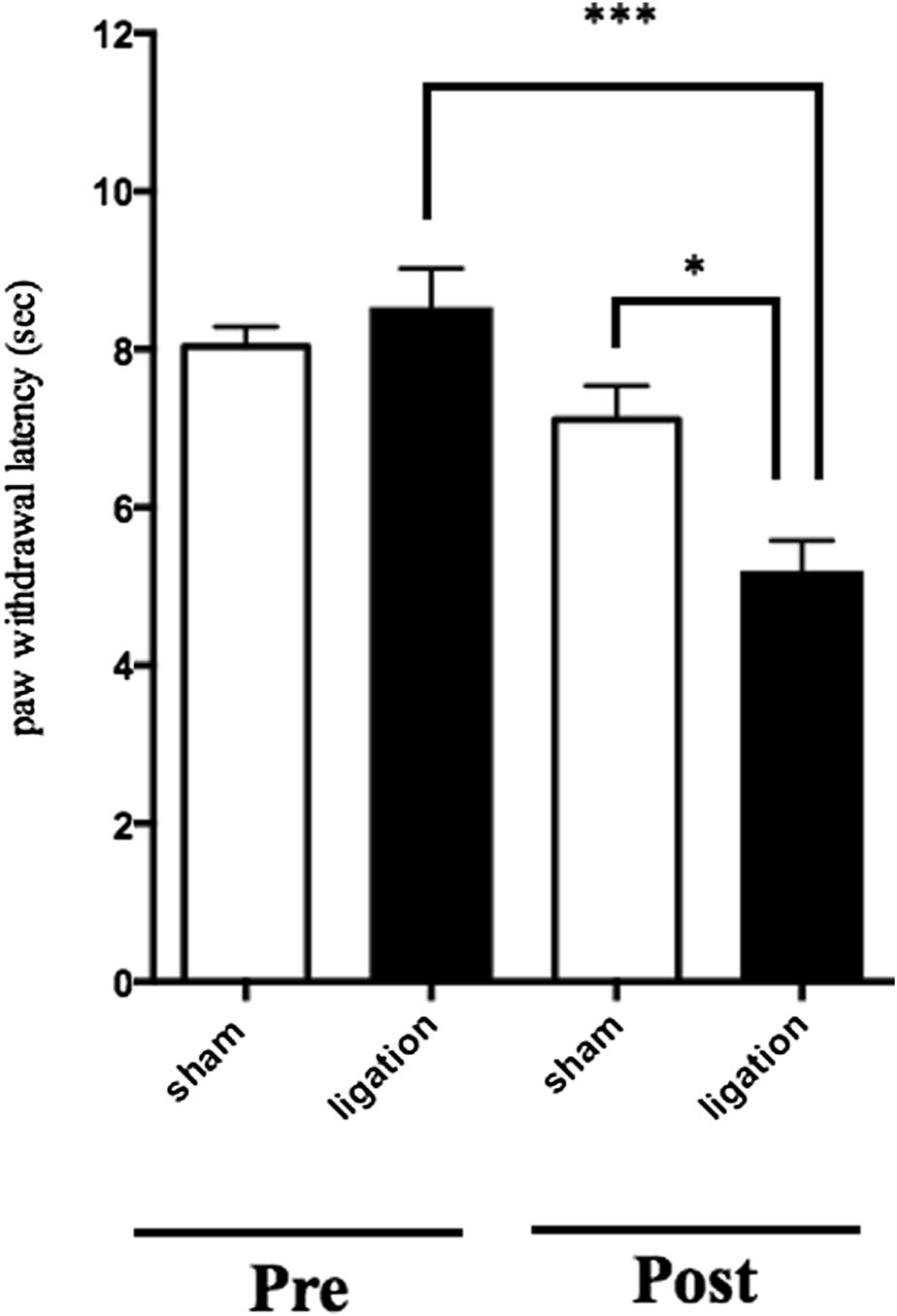
**Effect of sciatic nerve ligation on thermal hyperalgesia.** A plantar test was performed at 7 days after sciatic nerve ligation or sham operation. The statistical significance of differences between groups was assessed by one-way ANOVA. Each point represents the mean ± SEM (n = 8). *p < 0.05, ***p < 0.001 vs. sham.

Sciatic nerve ligation caused a marked decrease in the latency of paw withdrawal in response to thermal stimuli only on the ipsilateral side (Figure 1: thermal hyperalgesia, p <0 .05 vs. sham group).

### Changes in vigilance under a neuropathic pain-like state with EEG/EMG

**Figure 2:**
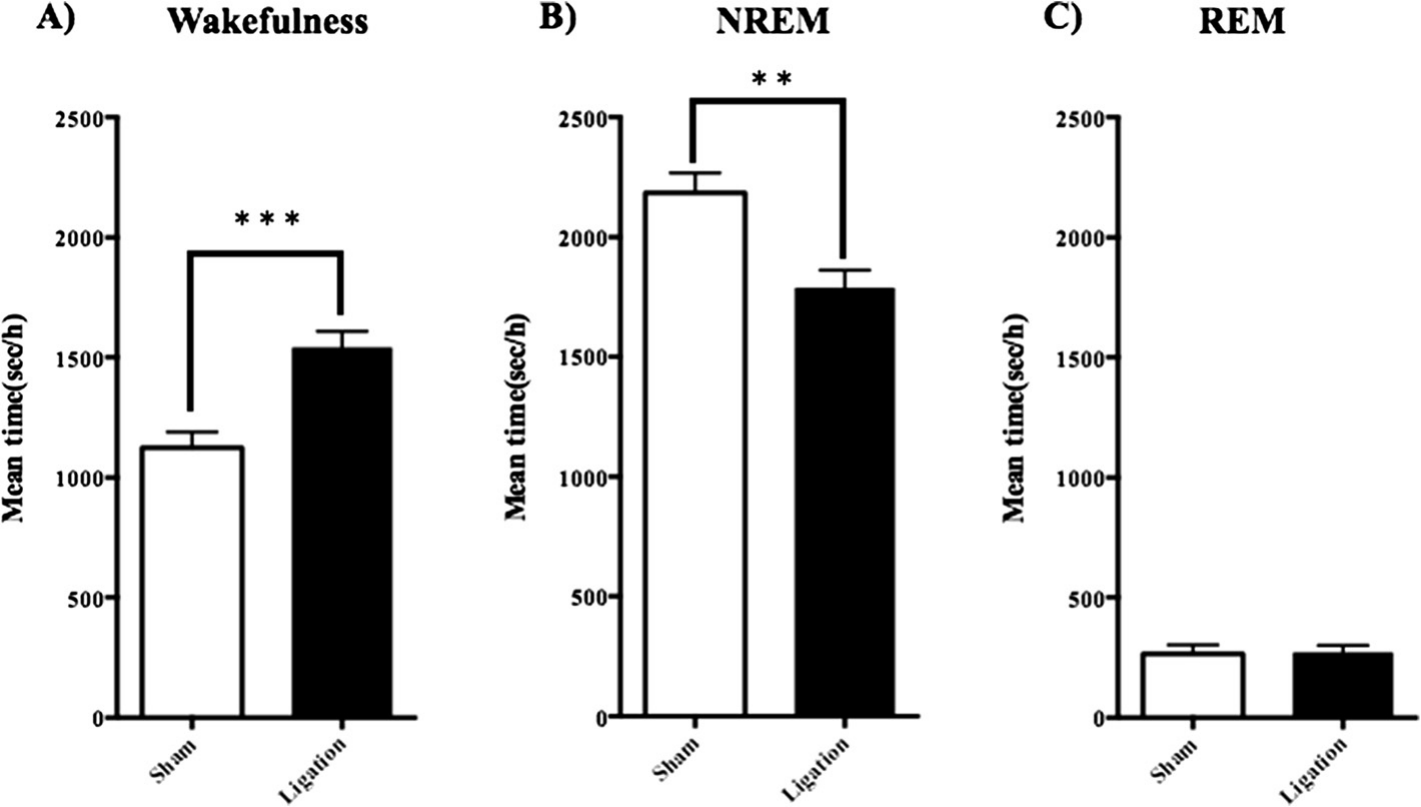
**Change in the sleep/wake pattern as determined by EEG/EMG at 7 days after sciatic nerve ligation.** The total mean times (sec/h) in wakefulness **(A)**, NREM sleep **(B)** and REM sleep **(C)** were determined by EEG/EMG recording for 24 hrs. Each point represents the mean ± SEM (n = 11). Student’s *t*-test was performed. *p < 0.05, **p < 0.01 and ***p < 0.001 compared with the sham group.

With this experimental model for neuropathic pain, we investigated the changes in sleep patterns. Frontal cortical activity and postural muscle tone, as monitored by EEG/EMG, are useful for identifying sleep/wake abnormalities. Vigilance was classified offline into 3 stages: wakefulness, rapid eye movement sleep, and NREM sleep. Sciatic nerve ligation for 7 days was associated with statistically significant increases in wakefulness (Figure 2A: p < 0.001 vs. sham group). Sciatic nerve ligation mice showed a statistically significant decrease in NREM sleep (Figure 2B: p < 0.01 vs. sham group). No difference in REM sleep was found between sham operation and sciatic nerve ligation for 7 days (Figure 2C).

### Distribution of fluoro-gold(FG)-labeled cells following microinjection in the PFC

**Figure 3:**
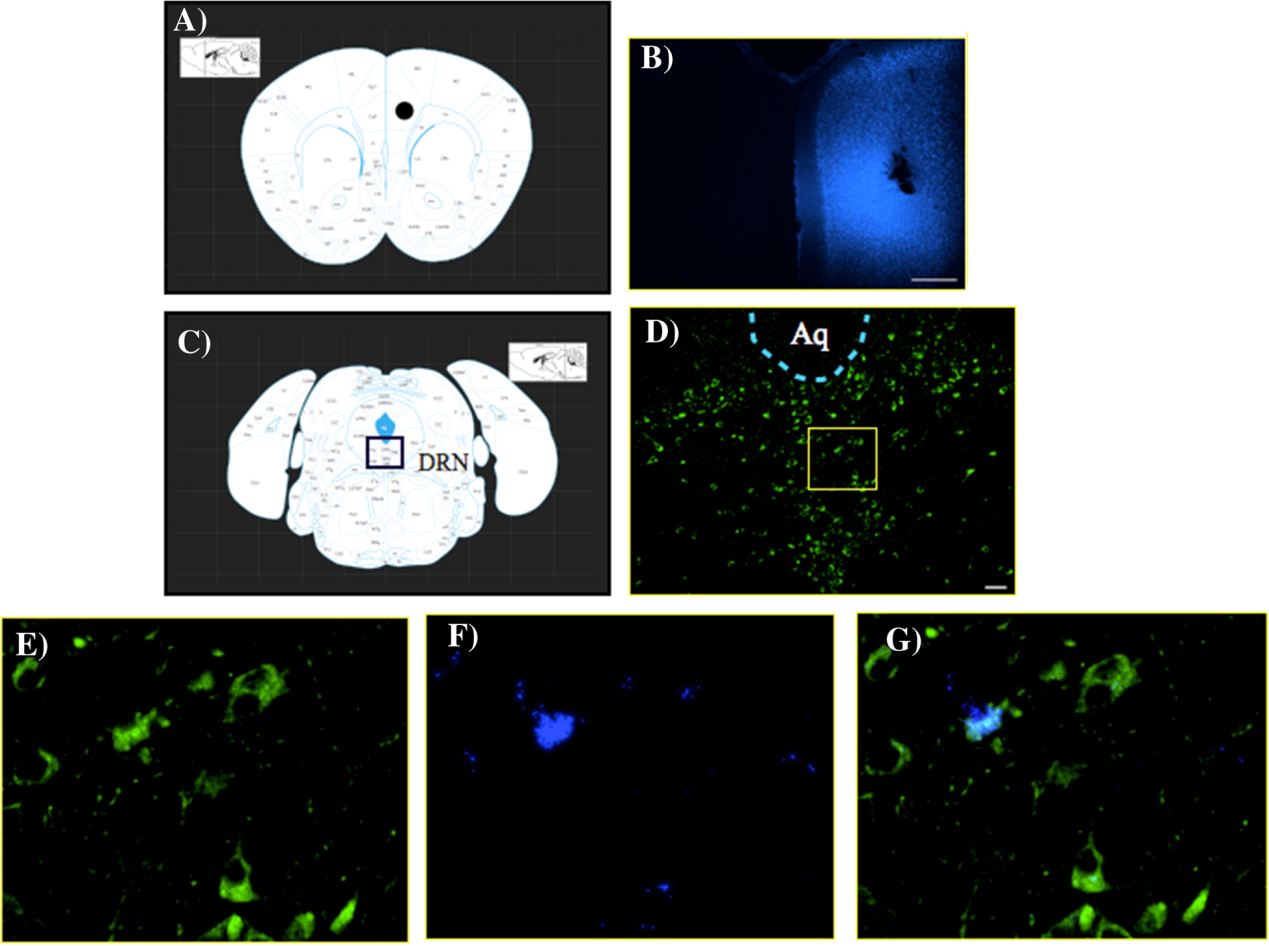
**Projection of 5-HT neurons from the dorsal raphe nucleus (DRN) to the prefrontal cortex (PFC) of mice.** Schematic illustrations of the injection site (symbols) in the PFC **(A)**. The image shows the extent of FG diffusion at the injection site **(B)**. Location of the DRN in a coronal section of mouse brain **(C)**. Tph2-immunoreactivity was noted in the DRN **(D, E)**. Cells in the DR after the microinjection of FG into the PFC **(F)**. Double-labeling experiments showed that FG-positive cells overlapped Tph2-positive cells in the DRN **(G)**. Scale bars = 50 μm **(B and D)**. Aq: aqueduct of the cerebrum.

To determine whether the DRN is linked to the PFC, which would permit possible functional influences on sleep and arousal neural circuits, we next investigated whether there were any neuronal projections from the DRN to the PFC using FG as a retrograde tracer. A schematic illustration of the injection site in the PFC is shown with a symbol (Figure 3A). Microinjection of FG into the region of the unilateral PFC produced a well-restricted injection site (Figure 3B). The DRN was shown to be located in a coronal section of the mouse brain (Figure 3C). Tryptophan hydroxylase 2 (Tph2)-labeled cells (Figure 3D, E) or FG-labeled cell bodies (Figure 3F) were apparently detected in the DRN after the microinjection of FG into the PFC. The population of retrogradely labeled neurons in the DRN also showed Tph2-immunoreactivity (Figure 3G).

### Change in the increase in dialysate 5-HT levels in the PFC induced by electrical stimulation of the DRN

**Figure 4:**
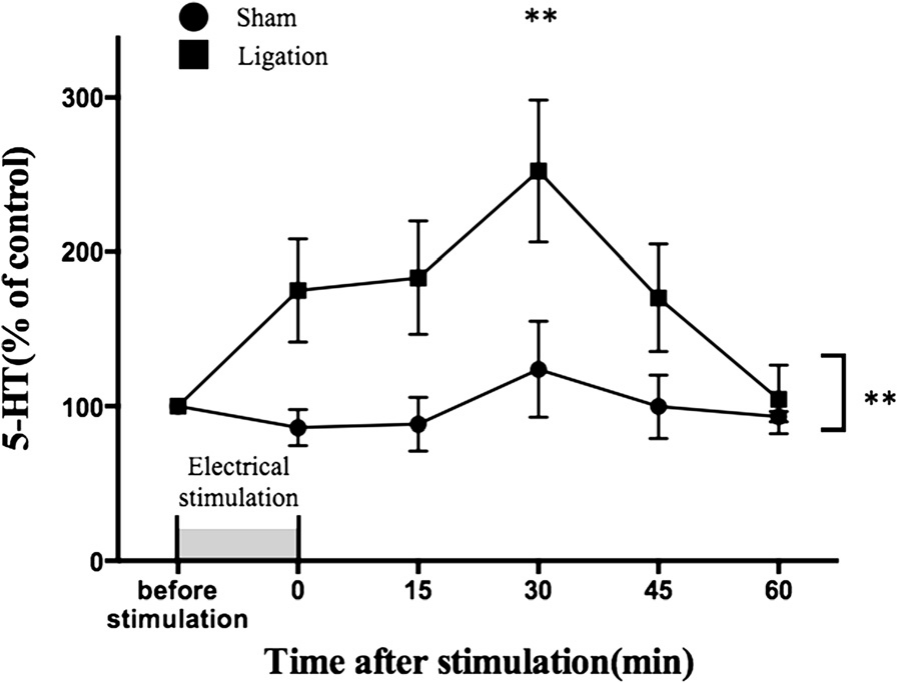
**Change in the dialysate levels of 5-HT induced by electrical stimulation in the mouse DRN at 7 days after sciatic nerve ligation.** To investigate the effect of electrical stimulation of the DRN, we evaluated the dialysate 5-HT levels in the PFC in mice by *in vivo* microdialysis. The mice were subjected to electrical stimulation, sine-wave pulses (100 Hz, 150 μA), for 15 min. The data are expressed as percentages of the corresponding baseline levels ± SEM (n = 4). **p < 0.01 vs. sham. The X-axis shows different time points after stimulation.

To investigate whether sciatic nerve ligation induces the hypersensitivity of DRN-PFC 5-HTergic neurons, we tested the effect of repetitive stimulation with rectangular electrical pulses for 15 min. In an *in vivo* microdialysis study, the dialysate 5-HT levels in the PFC of sciatic nerve-ligated mice were significantly increased by electrical stimulation (100 Hz, 150 μA) (Figure 4: p < 0.01 vs. sham group). Under the same conditions, there was no increase in the levels of dialysate 5-HT in the PFC of sham-operated mice.

### Expression of optogenetic transgene in DRN and PFC neurons of Tph2-ChR2 transgenic mice

**Figure 5:**
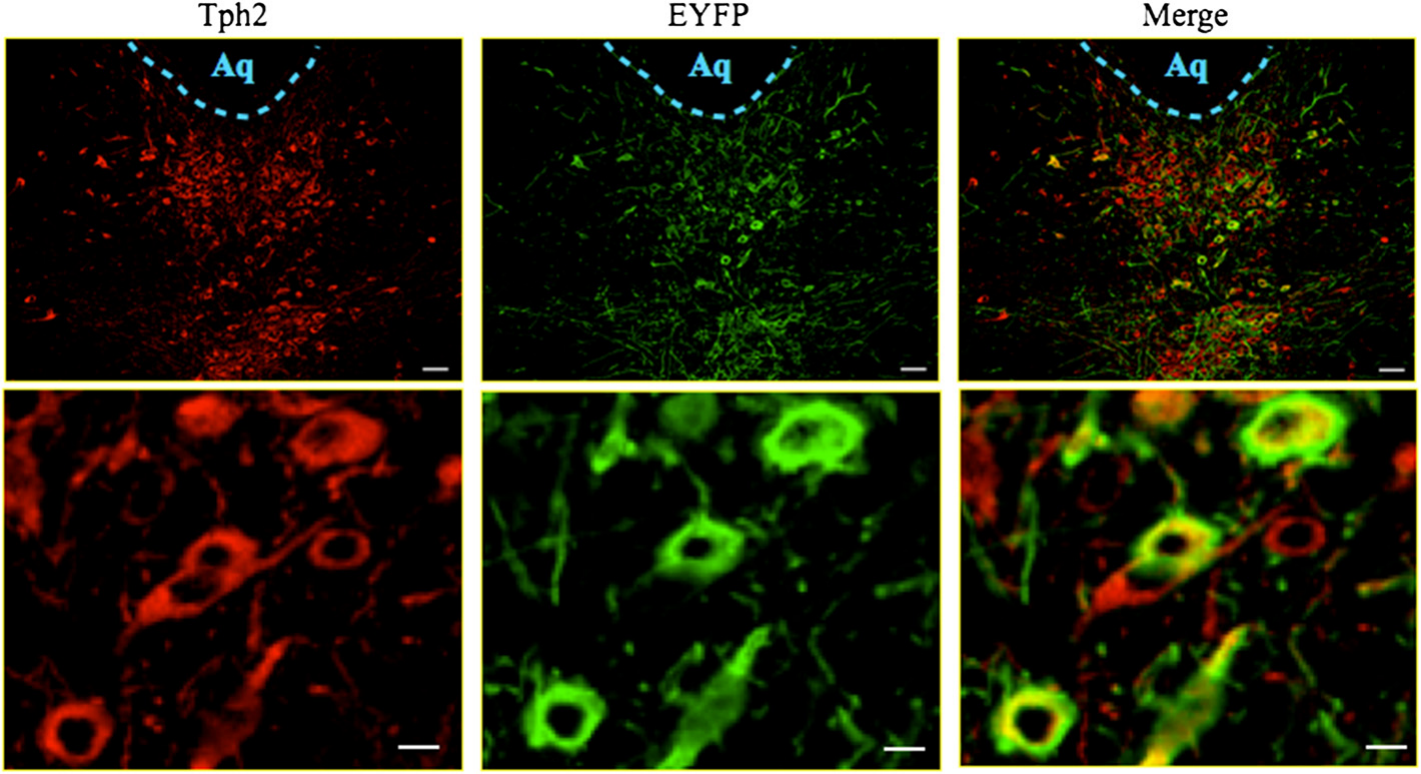
**Specific expression of optogenetic transgenes in the DRN.** Representative photomicrographs depicting Tph2 immunoreactivity (left column, red), EYFP immunoreactivity (center column, green) and merged images (right column) in the DRN obtained from Tph2-ChR2 transgenic mice. Top row, expression in the entire DRN (Scale bars, 50 μm); bottom row, individual neurons (Scale bars, 10 μm).

We investigated the expression of ChR2-EYFP in the DRN using immunohistochemistry. The DRN showed a high density of EYFP-immunoreactivity in slices of the mouse brainstem. To confirm the co-localization of ChR2-EYFP and Tph2 in the DRN, double-labeling experiments were performed. The results showed that Tph2-immunoreactivity was mostly co-localized with ChR2-EYFP (Figure 5).

### Change in the dialysate levels of 5-HT induced by optical stimulation in the mouse DRN

**Figure 6:**
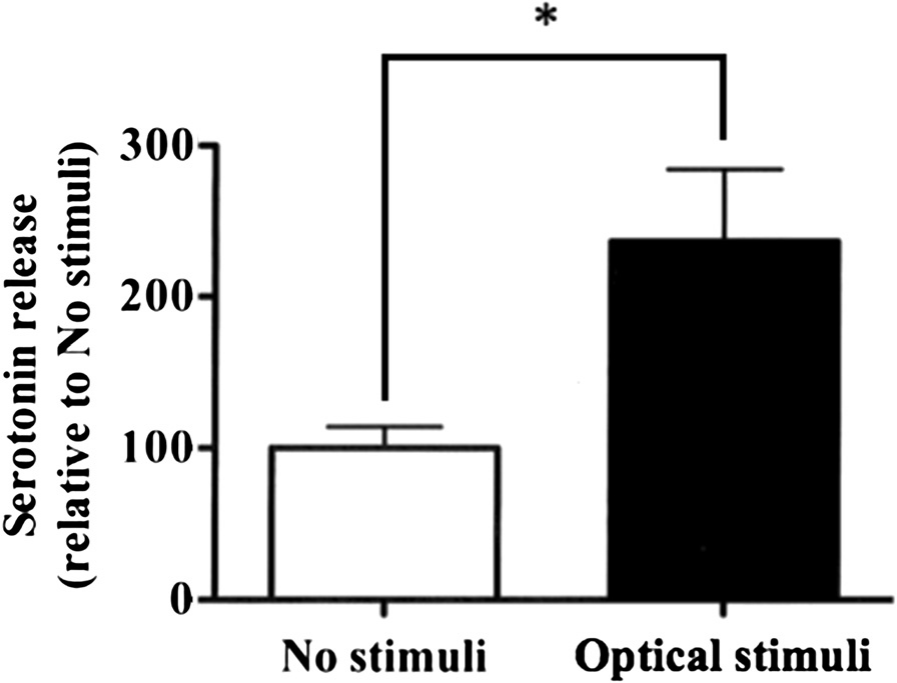
**Change in the dialysate levels of 5-HT induced by optical stimulation in the mouse DRN.** Extracellular 5-HT content in the PFC during optical stimulation at 20 Hz in the DRN. Values are shown as the means with SEM(n = 3). *p < 0.05 vs. No stimuli.

We confirmed that optical stimulation of the DRN affected the levels of 5-HT secreted from nerve terminals in the PFC. In an *in vivo* microdialysis study, the dialysate 5-HT levels in the PFC of Tph2-ChR2 transgenic mice were significantly increased by optical stimulation of the DRN (p < 0.05: vs. No stimuli, Figure 6).

### Optical stimulation of the DRN causes sleep-arousal transitions

**Figure 7:**
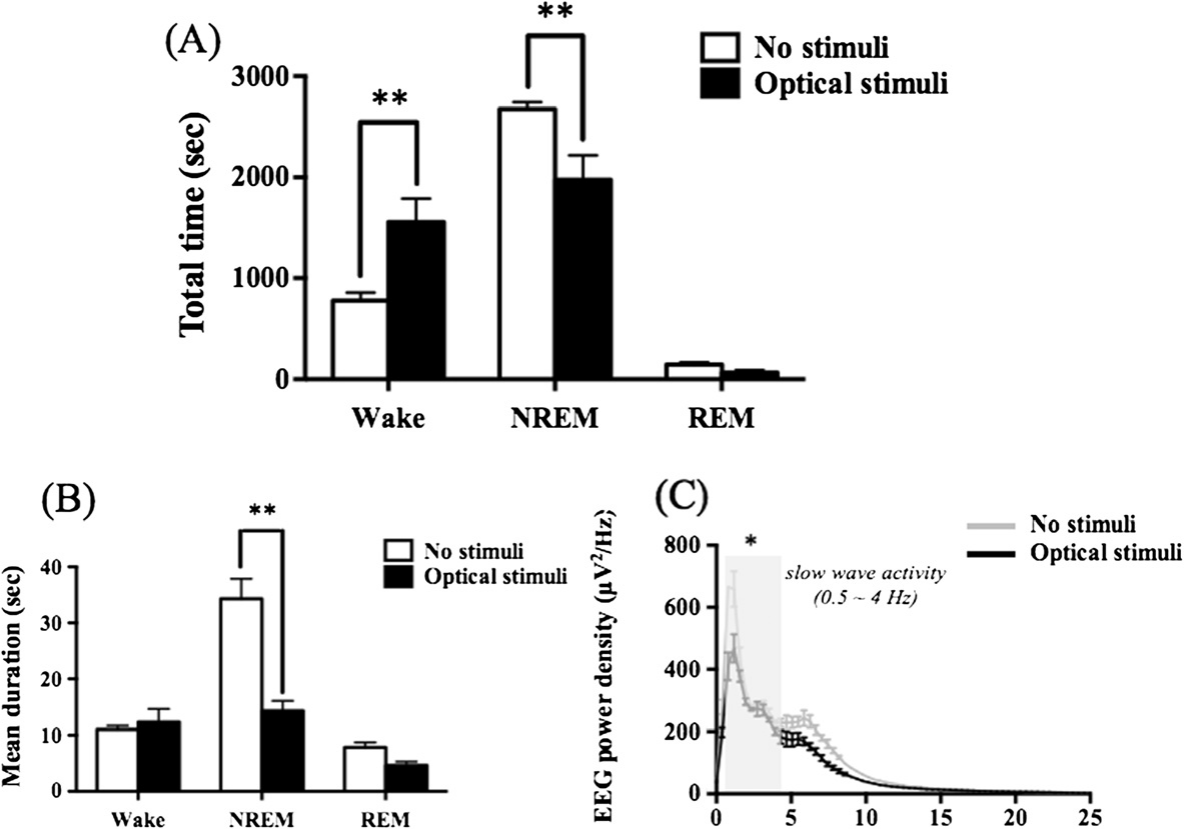
**Changes in the sleep/wake pattern induced by optical stimulation in the mouse DRN.** Mean time **(A)**, mean duration **(B)** and relative EEG power of NREM **(C)** were determined by EEG/EMG recordings for 3 hr. Values are shown as the means with SEM (n = 3). *p < 0.05 and **p < 0.01 vs. No stimuli.

To determine whether neural activation in DRN-5-HTergic neurons is necessary for interfering with sleep, we tested the effect of long-term optical stimulation of the DRN (8 mW, 20 Hz, 473 nm, blue light for 1 hr) on the sleep-wake architecture during inactive periods. Tph2-ChR2 transgenic mice showed a significant increase in wakefulness (p < 0.01 vs. No stimuli) and a significant decrease in NREM sleep (p < 0.01 vs. No stimuli) during optical stimulation, compared with no stimuli (Figure 7A). The duration of NREM sleep episodes was significantly decreased during optical stimulation in these mice (Figure 7B: p < 0.01 vs. No stimuli). During optical stimulation, there was a significant decrease in slow wave activity (0.5-4 Hz) in Tph2-ChR2 transgenic mice (p < 0.05: vs. No stimuli, Figure 7C).

## Discussion

Sciatic nerve constriction was previously reported to induce poor sleep quality in rats, particularly during the first week with this condition [24]. In our present study, we demonstrated that sciatic nerve-ligated mice exhibit dysregulation of the sleep/arousal pattern. These findings suggest that neuropathic pain induced by peripheral nerve injury may lead to the development of insomnia.

We analyzed the neural circuit using the retrograde tracer FG to investigate the nuclear origins of 5-HTergic neurons. We injected FG into the PFC, which is associated with sleep/arousal patterns [25]. FG-labeled cell bodies were detected in the DRN, and Tph2-immunoreactivity was revealed in a population of these labeled neurons. The DRN is one of the raphe nuclei located in the midline of the brainstem and is one of the largest 5-HTergic nuclei. Immunohistochemical studies have reported that the DRN contains as many as 9,200 5-HT neurons [26] or as few as 4,000 [27] in mice. Neurons from the DRN provide a substantial proportion of the 5-HT innervation to the forebrain through their widespread projections [28].

In a previous study, 5-HT transporter expression was shown to be significantly increased in the DRN of sciatic nerve-ligated rats [13]. However, few studies have investigated the direct influence of neuropathic pain on DRN 5-HTergic neurons. Therefore, we performed an *in vivo* microdialysis study to determine the activity of 5-HTergic neurons from the DRN to the PFC under a neuropathic pain-like state. The 5-HT level at the PFC was significantly increased in nerve-ligated mice compared with that in sham-operated mice after electrical stimulation of the DRN. These results suggest that neuropathic pain is associated with an increased activity of 5-HTergic neurons projecting from the DRN to the PFC.

Several areas in the brainstem and forebrain are important for modulation and expression of the sleep/wake cycle. It is now well established that several neurotransmitters and neuropeptides are involved in modulation of the sleep/wake cycle. 5-HT has been known for many years to play a role in the modulation of sleep. Early studies suggested that 5-HT is necessary to achieve and maintain behavioral sleep [3,29-31]. However, the 5-HT-sleep connection was strongly contradicted by data on the activity of the DRN, in that the activity of DRN neurons was greatest during wakefulness, considerably lower during NREM sleep, and completely abolished during REM sleep [4-9]. Also, the cooling of raphe neurons induced sleep [32,33]. Thus, it is still very controversial how and where 5-HT participates in this modulation.

A major advantage of an optogenetic approach versus traditional electrical stimulation [34] or pharmacological manipulation [35-38] is its ability to elicit neural activity at specifically defined temporal windows with minimal disturbance to the animal [39]. Electrical stimulation has a high probability of exerting excessive effects on other neurons and glial cells around the target, since the DRN in mice is quite small. On the other hand, the stimulation of DRN neurons using the local microinjection of pharmacological agents affects circumventricular organs due to the anatomical location of the DRN. In fact, it is possible for the injected agents to leak into the cerebral ventricles. The optogenetic gain-of-function approach has achieved not only regiospecific effects but also efficient genetic targeting of 5-HTergic neurons, which may explain the difference between the results of the optogenetic approach and those of other studies. In the present study, we therefore performed a system function analysis of DRN-5-HTergic neurons using transgenic mice that express ChR2 in neurons containing Tph2. We first performed an immunohistochemistry study to evaluate the expression of ChR2 in 5-HTergic neurons in the DRN. Although our immunostaining experiment on DRN neurons in acute brainstem slices showed ChR2 expression in DRN 5-HTergic neurons, we cannot be certain that these tools modulate neural activity as precisely *in vivo*. Therefore, we performed an *in vivo* microdialysis study to evaluate the levels of 5-HT released in the PFC during optical stimulation of the DRN, which increases the 5-HT level. Using this strategy, we confirmed the proper functioning of ChR2 expressed in the DRN. Next, we evaluated changes in the sleep and arousal pattern using EEG and EMG during optical stimulation of the DRN expressing ChR2. With optical stimulation, the total duration of wakefulness increased and that of NREM sleep decreased. These results suggest that the activation of DRN 5-HTergic neurons facilitates awakening. In contrast, activation of the DRN caused a decrease in the duration of the NREM sleep stage. Therefore, we conclude that the DRN is sufficient to promote sleep-to-wake transitions. Furthermore, in the power density of the EEG analysis, there was a significant decrease in slow wave activity during optical stimulation. This observation indicates that the activation of DRN-5-HTergic neurons has the potential to change the quality of sleep.

The functional role of 5-HT neurons in relation to the sleep/wake cycle may not only be confined to the DRN, but is also evident in other 5-HT sources of the brainstem [40], such as the median raphe nucleus, which gives rise to the majority of serotonergic ascending fibers into the forebrain [41-43]. Thus, further experiments on the influence of other 5-HT sources are needed to better understand the role of 5-HTergic networks in regulating the sleep-wake pattern under both physiological and chronic pain conditions.

Based on genetic [44-48] and pharmacological experiments [36,49-52], it is accepted that 5-HT functions predominantly to promote wakefulness. Although we demonstrated that both neuropathic pain and the specific activation of DRN-5HTergic neurons lead to a decrease in NREM sleep and no difference of REM sleep, the causal mechanism of this difference between REM and NREM is still unclear. In addition, it is unclear whether the enhanced release of 5-HT in the PFC is essential for neuropathic pain-induced sleep dysregulation, since our preliminary data show that no significant increase in the basal level of released 5-HT was likely to be seen without electrical stimulation when the sleep/wake pattern was changed (data not shown). We hypothesize that the reason why we did not observe an increase at this stage may be because under pathophysiological conditions, chronic pain may gradually increase the basal release of 5-HT in the PFC and affect the sleep/wake pattern, but constantly this will return to the normal level. Although we cannot completely exclude the possibility that the enhanced release of 5-HT in the PFC is not essential for chronic pain-induced abnormal sleep, we propose here that this activation in DRN neurons may, at least in part, be associated with sleep dysregulation under a neuropathic pain-like state. Furthermore, we also believe that other nuclei, such as the noradrenergic locus ceruleus nucleus [53], histaminergic tuberomammilary nucleus [54], cholinergic pedunculopontine nucleus and lateral tegmental nucleus [55,56], as well as multiple cell types in the basal forebrain [57], may also presumably contribute to sleep-to-wake transitions.

## Conclusion

In conclusion, we have demonstrated that neuropathic pain leads to sleep dysregulation, in parallel with the increased activity of DRN 5-HTergic neurons. Using an optogenetic approach, we found that the activation of DRN neurons facilitates sleep-to-wake transitions, which results in the dysregulation of sleep. Although further clarification is needed, we propose that acceleration of the activity of DRN-5-HTergic neurons may at least partly be associated with sleep dysregulation in a state of neuropathic pain.

## Methods

### Animals

The present study was conducted in accordance with the Guiding Principles for the Care and Use of Laboratory Animals at Hoshi University, as adopted by the Committee on Animal Research of Hoshi University (Tokyo, Japan). Male C57BL/6 J mice (8 weeks; Tokyo Laboratory Animals Science, Tokyo, Japan) and Tph2-ChR2 transgenic mice (B6; SJL-TG (Tph2-COP*H134R/EYFP) 5Gfng/J) [16] were used in this study. Tph2 is the rate-limiting enzyme in the synthesis of 5-HT in the central nervous system. The transgene ChR2 (H134R)-EYFP was constructed into the ATG (initiation codon) site of the first exon of Tph2. Animals were housed in a room maintained at 23 ± 1°C with a 12-hr light–dark cycle. Food and water were available *ad libitum.* Every effort was made to minimize the numbers and any suffering of animals used in the following experiments. Each animal was used only once.

### Neuropathic pain model

Mice were anesthetized with 3% isoflurane. We produced a partial sciatic nerve ligation model as described previously [58]. In brief, the sciatic nerve on the right side (ipsilateral side) was tied by a tight ligature with 8–0 silk suture around approximately one-half its diameter under a light microscope (SD30; Olympus, Tokyo, Japan). In sham-operated mice, the nerve was only exposed without ligation.

### Measurement of thermal thresholds

C57BL/6 J mice were used for the measurement of thermal thresholds. To assess the sensitivity to thermal stimulation, the right plantar surface of mice was tested individually by using a well-focused, radiant heat light source (model 33 Analgesia Meter; IITC/Life Science Instruments, California, USA). The intensity of the thermal stimulus was adjusted to achieve an average baseline paw-withdrawal latency of approximately 8–10 sec in naive mice. The paw-withdrawal latency was determined as the average of three measurements per paw. Only quick hind paw movements (with or without licking of hind paws) away from the stimulus were considered to be a withdrawal response. Paw movements associated with locomotion or weight-shifting were not counted as a response. The paws were measured alternating between the left and right with an interval of more than 3 min between measurements. Before the behavioral responses to the thermal stimulus were tested, mice were habituated for at least 1 hr in a clear acrylic cylinder (15 cm high and 8 cm in diameter). Under these conditions, the latency of paw withdrawal in response to the thermal stimulus was tested. The data represent the average value for the withdrawal latency of the right hind paw.

### EEG and EMG recordings

Under 3% isoflurane anesthesia, mice were implanted with EEG and EMG electrodes for polysomnographic recordings (Pinnacle Technology, Oregon, USA). Briefly, to monitor EEG signals, two stainless steel EEG recording screws were positioned 1 mm anterior to the bregma or lambda, both 1.5 mm lateral to the midline. EMG activity was monitored by stainless steel, Teflon-coated wires placed bilaterally into both trapezius muscles. Sleep–wake states were then monitored for a period of 24 hr at 2 days after placement of the EEG recording screws, encompassing both the baseline and the experimental day. The EEG/EMG signals were amplified, filtered (EEG, 0.5–30 Hz; EMG, 20–200 Hz), and digitized at a sampling rate of 128 Hz. The collected data were analyzed by software (SLEEPSIGN; Kissei Comtec, Nagano, Japan). Vigilance was automatically classified off-line under 5-sec epochs into three stages, i.e., wakefulness, REM and NREM sleep, by SLEEPSIGN according to the standard criteria. As a final step, defined sleep–wake stages were examined visually and corrected, if necessary. For each epoch, the EEG power density in the δwave (0.75–4.0 Hz) and θwave (6.25-9.0 Hz) and the integrated EMG value were displayed on a PC monitor. Three vigilance states -(1) waking (high EMG and low EEG amplitude and high θwave activity concomitant with the highest EMG values), (2) NREM sleep (low EMG and high EEG amplitude, high δwave activity), and (3) REM sleep (low EMG and low EEG amplitude, high θwave activity)- were determined for 5-sec epochs, and the scores were entered into a computer via a keyboard. EEG and EMG activities were monitored at 7 days after sciatic nerve ligation.

### Retrograde tracing study

First, C57BL/6 J mice were deeply anesthetized with 3% isoflurane. The anesthetized animals were placed in a stereotaxic apparatus. The skull was exposed, and a hole was drilled through the skull over the dorsolateral PFC, according to an atlas [59]. Micropipettes were filled with retrograde tracer, FG solution (Fluorochrome, Colorado, USA; 4% solution in saline), and 0.2 μl of the tracer was injected over 30 sec. The micropipette was left in place for 1 min after injection was complete to avoid leakage of the tracer along the pipette track, and then withdrawn from the brain. Two days after the injections, animals were perfused with formalin. The distribution of gold-emitting FG retrogradely labeled neurons and FG injection sites were detected using a microscope (BX-60; Olympus, Tokyo, Japan).

### Electrical stimulation

At the start of the surgical procedures, C57BL/6 J mice were anesthetized with 3% isoflurane and placed in a small animal stereotaxic apparatus. Mice underwent surgical implantation of a bipolar concentric electrode (MS3033SPCE; Bio Research, Nagoya, Japan) at least 1 day before the experiments. The electrode was placed in the DRN (from the bregma: anteroposterior, -4.9 mm; mediolateral, + 0 mm; dorsoventral, -3.4 mm) and fixed to the skull with quick self-curing acrylic resin. Electrical stimulation was controlled by an electronic stimulator (Nihon Kohden, Tokyo, Japan) to generate repetitive rectangular pulses (duration, 500 μs; intensity, 150 μA; frequency, 100 Hz).

### In vivo microdialysis and high-performance liquid chromatography

Under anesthesia with 3% isoflurane, mice were placed in a stereotaxic apparatus and a microdialysis probe (D-1-6-01; Eicom, Tokyo, Japan) was implanted directly into the PFC (from the bregma: anteroposterior, +1.5 mm; mediolateral, + 0.5 mm; dorsoventral, -3.7 mm) according to an atlas of the mouse brain [59]. The probe was fixed to the cranial bone with quick self-curing acrylic resin. More than 24 hr after surgery, the mice were placed in experimental cages. The probes were perfused continuously (1 μl/min) with artificial cerebrospinal fluid: 0.9 mM MgCl_2_, 147.0 mM NaCl, 4.0 mM KCl, and 1.2 mM CaCl_2_. Outflow fractions were collected every 15 min. After more than four baseline fractions were collected, mice were given electrical stimulation or optical stimulation for 15 min. Dialysis samples were collected for 1 hr after electrical stimulation or optical stimulation and analyzed by high-performance liquid chromatography with electrochemical detection (HTEC-500; Eicom, Tokyo, Japan). 5-HT was separated by column chromatography and identified and quantified by the use of a standard (Sigma Aldrich, Missouri, USA).

### Immunohistochemistry

Tph2-ChR2 transgenic mice were deeply anesthetized by the inhalation of 3% isoflurane with oxygen and perfusion-fixed with 4% paraformaldehyde in 0.1 M phosphate-buffered saline (pH 7.4). After perfusion, the brain was quickly removed and thick coronal sections of the frontal cortex including the cingulate cortex or the brainstem including the DRN were rapidly dissected and fixed in 4% paraformaldehyde for 2 hr. They were then permeated with 20% sucrose in 0.1 M phosphate-buffered saline for 1 day and 30% sucrose in 0.1 M phosphate-buffered saline for 2 days with agitation. The brain sections were finally frozen in an embedding compound (Sakura Finetechnical, Tokyo, Japan) on isopentane using liquid nitrogen and stored at -30°C. Transverse sections were cut with a cryostat (Leica CM1510; Leica Microsystems, Heidelberg, Germany) at a thickness of 8 μm and thaw-mounted on poly-L-lysine-coated glass slides. Brain sections including PFC or DRN were incubated in blocking solution, 3% normal goat serum (Vector Laboratories, California, USA) in 0.01 M phosphate-buffered saline, for 1 hr at room temperature, and then incubated for 48 hr at 4°C with primary antibodies diluted in 3% normal goat serum: anti-Tph2 (mouse monoclonal.; Sigma Aldrich, Missouri, USA) and anti-green fluorescent protein (GFP; rabbit polyclonal; Molecular Probes, Oregon, USA) for double-staining. The antibody was then rinsed with phosphate-buffered saline and incubated with an appropriate secondary antibody conjugated with Alexa™ 546 (Molecular Probes, Oregon, USA) for 2 hr at room temperature. Fluorescence of immunolabelling was detected using a light microscope (BX61; Olympus, Tokyo, Japan) and digitized images.

### Optical stimulation

At the start of the surgical procedures, Tph2-ChR2 transgenic mice were anesthetized with 3% isoflurane and placed on a small animal stereotaxic apparatus. Mice underwent the surgical implantation of an 8 mm unilateral cannula (EIM-300; Eicom, Tokyo, Japan) at least 1 day before the experiments. The cannula was placed above the DRN and fixed to the skull with quick self-curing acrylic resin. An optical fiber (500 μm diameter; Lucir, Osaka, Japan) was placed inside the implanted cannula at least 2 hr before the experiments. The light source was a 473 nm blue laser (COME2-LB473 model; Lucir, Osaka, Japan) controlled by an electronic stimulator (Nihon Kohden, Tokyo, Japan) to generate tonic light pulses (duration, 10 ms; frequency, 20 Hz; intensity, 9 mW). The power output was measured at the tip of the fiber with a NOVA light meter (Ophir, Saitama, Japan) when the laser was activated in continuous mode.

### Statistical analysis

Data are expressed as the mean with SEM. The statistical significance of differences between the groups was assessed with one-way ANOVA or two-way ANOVA followed by the Bonferroni multiple comparisons test or unpaired-Student’s *t* test as appropriate. All statistical analyses were performed with Prism version 5.0a or 6.0b (GraphPad Software, California, USA).

## Abbreviations

ChR2: Channel rhodopsin 2; DRN: Dorsal raphe nucleus; EEG: Electroencephalogram; EMG: Electromyogram; FG: Fluoro-gold; GFP: Green fluorescent protein; NREM: Non rapid eye movement; PFC: Prefrontal cortex; REM: Rapid eye movement; Tph2: Tryptophan hydroxylase-2; 5-HT: 5-hydroxytryptamine (Serotonin).

## Competing interests

The authors declare that they have no competing financial interests.

## Authors’ contributions

HI performed experiments, analyzed data and wrote the first draft of the manuscript. MY designed experiments, performed experiments and analyzed data. AY and MN designed and performed experiments. CK, AH and YS performed experiments. DI, HS and MY wrote the paper. MN conceptualized the hypothesis, supervised research and finalized the manuscript. All authors read and approved the final manuscript.

## References

[B1] Smith MT Perlis ML Smith MS Giles DE Carmody TP Sleep quality and presleep arousal in chronic pain J Behav Med 2000 6 1 1 13 10.1023/A:1005444719169 10749008

[B2] Morin CM Gibson D Wade J Self-reported sleep and mood disturbance in chronic pain patients Clin J Pain 1998 6 4 311 314 10.1097/00002508-199812000-00007 9874009

[B3] Jouvet M The role of monoamines and acetylcholine-containing neurons in the regulation of the sleep-waking cycle Ergeb Physiol 1972 6 166 307 4403272 10.1007/3-540-05462-6_2

[B4] Trulson ME Jacobs BL Raphe unit activity in freely moving cats: correlation with level of behavioral arousal Brain Res 1979 6 1 135 150 10.1016/0006-8993(79)90157-4 218676

[B5] Cespuglio R Faradji H Gomez ME Jouvet M Single unit recordings in the nuclei raphe dorsalis and magnus during the sleep-waking cycle of semi-chronic prepared cats Neurosci Lett 1981 6 2 133 138 10.1016/0304-3940(81)90236-6 7254710

[B6] Lydic R McCarley RW Hobson JA Serotonin neurons and sleep. II. Time course of dorsal raphe discharge, PGO waves, and behavioral states Arch Ital Biol 1987 6 1 1 28 3449005

[B7] McGinty DJ Harper RM Dorsal raphe neurons: depression of firing during sleep in cats Brain Res 1976 6 3 569 575 10.1016/0006-8993(76)90480-7 1244990

[B8] Puizillout JJ Gaudin-Chazal G Daszuta A Seyfritz N Ternaux JP Release of endogenous serotonin from “encephale isole” cats. II - Correlations with raphe neuronal activity and sleep and wakefulness J Physiol Paris 1979 6 5 531 537 533869

[B9] Sakai K Sleep-waking discharge profiles of dorsal raphe nucleus neurons in mice Neuroscience 2011 6 200 224 21958868 10.1016/j.neuroscience.2011.09.024

[B10] Sanders KH Klein CE Mayor TE Heym C Handwerker HO Differential effects of noxious and non-noxious input on neurones according to location in ventral periaqueductal grey or dorsal raphe nucleus Brain Res 1980 6 1 83 97 10.1016/0006-8993(80)90257-7 7357452

[B11] Porro CA Cavazzuti M Galetti A Sassatelli L Functional activity mapping of the rat brainstem during formalin-induced noxious stimulation Neuroscience 1991 6 2–3 667 680 1870704 10.1016/0306-4522(91)90358-u

[B12] Dai JL Zhu YH Li XY Huang DK Xu SF C-fos expression during electroacupuncture analgesia in rats–an immunohistochemical study Acupunct Electrother Res 1992 6 3 165 176 1357923 10.3727/036012992816357738

[B13] Rojo ML Rodriguez-Gaztelumendi A Pazos A Diaz A Differential adaptive changes on serotonin and noradrenaline transporters in a rat model of peripheral neuropathic pain Neurosci Lett 2012 6 2 181 186 10.1016/j.neulet.2012.03.050 22480692

[B14] Deisseroth K Optogenetics Nat Methods 2011 6 1 26 29 21191368 10.1038/nmeth.f.324 PMC6814250

[B15] Deisseroth K Feng G Majewska AK Miesenbock G Ting A Schnitzer MJ Next-generation optical technologies for illuminating genetically targeted brain circuits J Neurosci 2006 6 41 10380 10386 10.1523/JNEUROSCI.3863-06.2006 17035522 PMC2820367

[B16] Zhao S Ting JT Atallah HE Qiu L Tan J Gloss B Augustine GJ Deisseroth K Luo M Graybiel AM Cell type–specific channelrhodopsin-2 transgenic mice for optogenetic dissection of neural circuitry function Nat Methods 2011 6 9 745 752 10.1038/nmeth.1668 21985008 PMC3191888

[B17] Boyden ES Zhang F Bamberg E Nagel G Deisseroth K Millisecond-timescale, genetically targeted optical control of neural activity Nat Neurosci 2005 6 9 1263 1268 10.1038/nn1525 16116447

[B18] Yizhar O Fenno LE Davidson TJ Mogri M Deisseroth K Optogenetics in neural systems Neuron 2011 6 1 9 34 10.1016/j.neuron.2011.06.004 21745635

[B19] Fenno L Yizhar O Deisseroth K The development and application of optogenetics Annu Rev Neurosci 2011 6 389 412 10.1146/annurev-neuro-061010-113817 21692661 PMC6699620

[B20] Zhang F Vierock J Yizhar O Fenno LE Tsunoda S Kianianmomeni A Prigge M Berndt A Cushman J Polle J The microbial opsin family of optogenetic tools Cell 2011 6 7 1446 1457 10.1016/j.cell.2011.12.004 22196724 PMC4166436

[B21] Aravanis AM Wang LP Zhang F Meltzer LA Mogri MZ Schneider MB Deisseroth K An optical neural interface: in vivo control of rodent motor cortex with integrated fiberoptic and optogenetic technology J Neural Eng 2007 6 3 S143 S156 10.1088/1741-2560/4/3/S02 17873414

[B22] Kravitz AV Kreitzer AC Optogenetic manipulation of neural circuitry in vivo Curr Opin Neurobiol 2011 6 3 433 439 10.1016/j.conb.2011.02.010 21420852 PMC3130851

[B23] Lima SQ Miesenbock G Remote control of behavior through genetically targeted photostimulation of neurons Cell 2005 6 1 141 152 10.1016/j.cell.2005.02.004 15820685

[B24] Andersen ML Tufik S Sleep patterns over 21-day period in rats with chronic constriction of sciatic nerve Brain Res 2003 6 1–2 84 92 12932842 10.1016/s0006-8993(03)03095-6

[B25] Rueter LE Jacobs BL Changes in forebrain serotonin at the light–dark transition: correlation with behaviour Neuroreport 1996 6 5 1107 1111 10.1097/00001756-199604100-00031 8804061

[B26] Daszuta A Portalier P Distribution and quantification of 5-HT nerve cell bodies in the nucleus raphe dorsalis area of C57BL and BALBc mice. Relationship between anatomy and biochemistry Brain Res 1985 6 1–2 58 64 3000536 10.1016/0006-8993(85)91220-x

[B27] Ishimura K Takeuchi Y Fujiwara K Tominaga M Yoshioka H Sawada T Quantitative analysis of the distribution of serotonin-immunoreactive cell bodies in the mouse brain Neurosci Lett 1988 6 3 265 270 10.1016/0304-3940(88)90691-X 3185964

[B28] Vertes RP A PHA-L analysis of ascending projections of the dorsal raphe nucleus in the rat J Comp Neurol 1991 6 4 643 668 10.1002/cne.903130409 1783685

[B29] Koella WP Czicman J Mechanism of the EEG-synchronizing action of serotonin Am J Physiol 1966 6 4 926 934 5926579 10.1152/ajplegacy.1966.211.4.926

[B30] Ursin R Does para-chlorophenylalanine produce disturbed waking, disturbed sleep or activation by ponto-geniculo-occipital waves in cats? Waking Sleeping 1980 6 3 211 221 6456599

[B31] Ursin R Differential effect of para-chlorophenylalanine on the two slow wave sleep stages in the cat Acta Physiol Scand 1972 6 2 278 285 10.1111/j.1748-1716.1972.tb05333.x 4344858

[B32] Cespuglio R Gomez ME Walker E Jouvet M Effect of cooling and electrical stimulation of nuclei of raphe system on states of alertness in cat Electroencephalogr Clin Neurophysiol 1979 6 3 289 308 10.1016/0013-4694(79)90281-5 90600

[B33] Cespuglio R Walker E Gomez ME Musolino R Cooling of the nucleus raphe dorsalis induces sleep in the cat Neurosci Lett 1976 6 4 221 227 10.1016/0304-3940(76)90077-X 19604890

[B34] Jha SK Ross RJ Morrison AR Sleep-related neurons in the central nucleus of the amygdala of rats and their modulation by the dorsal raphe nucleus Physiol Behav 2005 6 4 415 426 10.1016/j.physbeh.2005.06.033 16137725

[B35] Portas CM Thakkar M Rainnie D McCarley RW Microdialysis perfusion of 8-hydroxy-2-(di-n-propylamino)tetralin (8-OH-DPAT) in the dorsal raphe nucleus decreases serotonin release and increases rapid eye movement sleep in the freely moving cat J Neurosci 1996 6 8 2820 2828 8786456 10.1523/JNEUROSCI.16-08-02820.1996 PMC6578745

[B36] Monti JM Jantos H Differential effects of the 5-HT1A receptor agonist flesinoxan given locally or systemically on REM sleep in the rat Eur J Pharmacol 2003 6 2–3 121 130 14575796 10.1016/j.ejphar.2003.08.039

[B37] Bradley PB Marley E Effect of tryptamine and tryptamine homologues on cerebral electrical activity and behaviour in the cat Br J Pharmacol Chemother 1965 6 659 674 10.1111/j.1476-5381.1965.tb01622.x 14340920 PMC1704018

[B38] Adrien J Tissier MH Lanfumey L Haj-Dahmane S Jolas T Franc B Hamon M Central action of 5-HT3 receptor ligands in the regulation of sleep-wakefulness and raphe neuronal activity in the rat Neuropharmacology 1992 6 6 519 529 10.1016/0028-3908(92)90183-P 1407392

[B39] Tye KM Deisseroth K Optogenetic investigation of neural circuits underlying brain disease in animal models Nat Rev Neurosci 2012 6 4 251 266 10.1038/nrn3171 22430017 PMC6682316

[B40] Portas CM Bjorvatn B Ursin R Serotonin and the sleep/wake cycle: special emphasis on microdialysis studies Prog Neurobiol 2000 6 1 13 35 10.1016/S0301-0082(98)00097-5 10622375

[B41] Jacobs BL Fornal CA Activity of brain serotonergic neurons in the behaving animal Pharmacol Rev 1991 6 4 563 578 1775508

[B42] Adell A Celada P Abellan MT Artigas F Origin and functional role of the extracellular serotonin in the midbrain raphe nuclei Brain Res Brain Res Rev 2002 6 2–3 154 180 12423765 10.1016/s0165-0173(02)00182-0

[B43] Lechin F van der Dijs B Hernandez-Adrian G Dorsal raphe vs. median raphe serotonergic antagonism. Anatomical, physiological, behavioral, neuroendocrinological, neuropharmacological and clinical evidences: relevance for neuropharmacological therapy Prog Neuropsychopharmacol Biol Psychiatry 2006 6 4 565 585 10.1016/j.pnpbp.2005.11.025 16436311

[B44] Boutrel B Franc B Hen R Hamon M Adrien J Key role of 5-HT1B receptors in the regulation of paradoxical sleep as evidenced in 5-HT1B knock-out mice J Neurosci 1999 6 8 3204 3212 10191333 10.1523/JNEUROSCI.19-08-03204.1999 PMC6782285

[B45] Boutrel B Monaca C Hen R Hamon M Adrien J Involvement of 5-HT1A receptors in homeostatic and stress-induced adaptive regulations of paradoxical sleep: studies in 5-HT1A knock-out mice J Neurosci 2002 6 11 4686 4692 12040075 10.1523/JNEUROSCI.22-11-04686.2002 PMC6758830

[B46] Frank MG Stryker MP Tecott LH Sleep and sleep homeostasis in mice lacking the 5-HT2c receptor Neuropsychopharmacology 2002 6 5 869 873 10.1016/S0893-133X(02)00353-6 12431861 PMC2452994

[B47] Popa D Lena C Fabre V Prenat C Gingrich J Escourrou P Hamon M Adrien J Contribution of 5-HT2 receptor subtypes to sleep-wakefulness and respiratory control, and functional adaptations in knock-out mice lacking 5-HT2A receptors J Neurosci 2005 6 49 11231 11238 10.1523/JNEUROSCI.1724-05.2005 16339018 PMC6725907

[B48] Hedlund PB Huitron-Resendiz S Henriksen SJ Sutcliffe JG 5-HT7 receptor inhibition and inactivation induce antidepressantlike behavior and sleep pattern Biol Psychiatry 2005 6 10 831 837 10.1016/j.biopsych.2005.05.012 16018977

[B49] Monti JM Jantos H Effects of the serotonin 5-HT2A/2C receptor agonist DOI and of the selective 5-HT2A or 5-HT2C receptor antagonists EMD 281014 and SB-243213, respectively, on sleep and waking in the rat Eur J Pharmacol 2006 6 1–3 163 170 17059817 10.1016/j.ejphar.2006.09.027

[B50] Monti JM Jantos H Lagos P Activation of serotonin 5-HT(1B) receptor in the dorsal raphe nucleus affects REM sleep in the rat Behav Brain Res 2010 6 1 8 16 10.1016/j.bbr.2009.08.037 19737581

[B51] Monti JM Jantos H Monti D Increased REM sleep after intra-dorsal raphe nucleus injection of flesinoxan or 8-OHDPAT: prevention with WAY 100635 Eur Neuropsychopharmacol 2002 6 1 47 55 10.1016/S0924-977X(01)00133-X 11788240

[B52] Bjorvatn B Ursin R Effects of the selective 5-HT1B agonist, CGS 12066B, on sleep/waking stages and EEG power spectrum in rats J Sleep Res 1994 6 2 97 105 10.1111/j.1365-2869.1994.tb00112.x 10607113

[B53] Berridge CW Waterhouse BD The locus coeruleus-noradrenergic system: modulation of behavioral state and state-dependent cognitive processes Brain Res Brain Res Rev 2003 6 1 33 84 10.1016/S0165-0173(03)00143-7 12668290

[B54] Parmentier R Ohtsu H Djebbara-Hannas Z Valatx JL Watanabe T Lin JS Anatomical, physiological, and pharmacological characteristics of histidine decarboxylase knock-out mice: evidence for the role of brain histamine in behavioral and sleep-wake control J Neurosci 2002 6 17 7695 7711 12196593 10.1523/JNEUROSCI.22-17-07695.2002 PMC6757981

[B55] Steriade M Acetylcholine systems and rhythmic activities during the waking–sleep cycle Prog Brain Res 2004 6 179 196 14650916 10.1016/S0079-6123(03)45013-9

[B56] Boucetta S Jones BE Activity profiles of cholinergic and intermingled GABAergic and putative glutamatergic neurons in the pontomesencephalic tegmentum of urethane-anesthetized rats J Neurosci 2009 6 14 4664 4674 10.1523/JNEUROSCI.5502-08.2009 19357291 PMC6665745

[B57] Hassani OK Lee MG Henny P Jones BE Discharge profiles of identified GABAergic in comparison to cholinergic and putative glutamatergic basal forebrain neurons across the sleep-wake cycle J Neurosci 2009 6 38 11828 11840 10.1523/JNEUROSCI.1259-09.2009 19776269 PMC2790860

[B58] Malmberg AB Chen C Tonegawa S Basbaum AI Preserved acute pain and reduced neuropathic pain in mice lacking PKCgamma Science 1997 6 5336 279 283 10.1126/science.278.5336.279 9323205

[B59] Franklin K Paxinos G The Mouse Brain in Stereotaxic Coordinates 2007 San Diego: Academic

